# Characteristics of people living in Italy after a cancer diagnosis in 2010 and projections to 2020

**DOI:** 10.1186/s12885-018-4053-y

**Published:** 2018-02-09

**Authors:** Stefano Guzzinati, Saverio Virdone, Roberta De Angelis, Chiara Panato, Carlotta Buzzoni, Riccardo Capocaccia, Silvia Francisci, Anna Gigli, Manuel Zorzi, Giovanna Tagliabue, Diego Serraino, Fabio Falcini, Claudia Casella, Antonio Giampiero Russo, Fabrizio Stracci, Bianca Caruso, Maria Michiara, Anna Luisa Caiazzo, Marine Castaing, Stefano Ferretti, Lucia Mangone, Giuseppa Rudisi, Flavio Sensi, Guido Mazzoleni, Fabio Pannozzo, Rosario Tumino, Mario Fusco, Paolo Ricci, Gemma Gola, Adriano Giacomin, Francesco Tisano, Giuseppa Candela, Anna Clara Fanetti, Filomena Pala, Antonella Sutera Sardo, Massimo Rugge, Laura Botta, Luigino Dal Maso

**Affiliations:** 1 Veneto Tumor Registry, Veneto Region, Padova, Passaggio Gaudenzio 1, 35131 Padova, Italy; 2 Cancer Epidemiology Unit, CRO Aviano National Cancer Institute IRCCS, Via Franco Gallini 2, 33081 Aviano, PN Italy; 3 Istituto Superiore di Sanità (ISS), Rome, Italy; 4 Tuscany Cancer Registry, Clinical and Descriptive Epidemiology Unit, Cancer Prevention and Research Institute (ISPO), Florence, Italy; 5 AIRTUM Database, Florence, Italy; 6 Dipartimento di Ricerca Epidemiologica e Medicina Molecolare (DREaMM), Fondazione IRCCS Istituto Nazionale dei Tumori, Milan, Italy; 7 Institute for Research on Population and Social Policies, National Research Council, Rome, Italy; 8 Lombardy Cancer Registry, Varese Province, Cancer Registry Unit, Department of Research, Fondazione IRCCS Istituto Nazionale dei Tumori, Milan, Italy; 9 Romagna Cancer Registry, Istituto Scientifico Romagnolo per lo Studio e la Cura dei Tumori (IRST) IRCCS, Meldola (Forlì), Italy-Azienda Usl della Romagna, Forlì, Italy; 10 Registro Tumori Ligure, Epidemiologia Clinica, Ospedale Policlinico San Martino IRCCS, Genova, Italy; 11 Cancer Registry of Milan, Epidemiology Unit, Agency for Health Protection of Milan, Milan, Italy; 12 Public Health Section, Department of Experimental Medicine, University of Perugia, Perugia, Italy; 13 Modena Cancer Registry, Public Health Department, AUSL Modena, Modena, Italy; 14 Parma Cancer Registry, Oncology Unit, Azienda Ospedaliera Universitaria di Parma, Parma, Italy; 15 Cancer Registry of Salerno Province, Salerno, Italy; 16 Registro Tumori Integrato Catania-Messina-Siracusa-Enna, Università degli Studi di Catania, Catania, Italy; 17 Ferrara Cancer Registry, Ferrara Local Health Board, University of Ferrara, USL Ferrara, Ferrara, Italy; 18 Reggio Emilia Cancer Registry, Epidemiology unit, AUSL ASMN-IRCCS, Azienda USL di Reggio Emilia, Reggio Emilia, Italy; 19 Palermo and Province Cancer Registry, Clinical Epidemiology Unit, Azienda Ospedaliera Universitaria Policlinico “Paolo Giaccone”, Palermo, Italy; 20 North Sardinia Cancer Registry, Azienda Regionale per la Tutela della Salute, Sassari, Italy; 21 Sudtyrol Cancer Registry, Bolzano, Italy; 22 Cancer Registry of Latina Province, AUSL Latina, Latina, Italy; 23 Cancer Registry ASP Ragusa, Ragusa, Italy; 24 Cancer Registry of ASL Napoli 3 Sud, Napoli, Italy; 25 Mantova Cancer Registry, Epidemilogy Unit, Agenzia di Tutela della Salute (ATS) della Val Padana, Mantova, Italy; 26 Como Cancer Registry, ATS Insubria, Varese, Italy; 27 Registro Tumori Piemonte, Provincia di Biella CPO, Biella, Italy; 28 Cancer Registry of of the Province of Siracusa, Local Health Unit of Siracusa, Siracusa, Italy; 29 Trapani Cancer Registry, Dipartimento di Prevenzione della Salute, Trapani, Italy; 30 Sondrio Cancer Registry, Health Protection Agency, Sondrio, Italy; 31 Nuoro Cancer Registry, RT Nuoro, ASSL Nuoro/ATS Sardegna, Nuoro, Italy; 32 Catanzaro Cancer Registry, Azienda Sanitaria 7, Catanzaro, Italy; 33 Department of Medicine (DIMED), University of Padua, Padua, Italy

**Keywords:** Cancer prevalence, Projections, Survivors, Italy

## Abstract

**Background:**

Estimates of cancer prevalence are widely based on limited duration, often including patients living after a cancer diagnosis made in the previous 5 years and less frequently on complete prevalence (i.e., including all patients regardless of the time elapsed since diagnosis). This study aims to provide estimates of complete cancer prevalence in Italy by sex, age, and time since diagnosis for all cancers combined, and for selected cancer types. Projections were made up to 2020, overall and by time since diagnosis.

**Methods:**

Data were from 27 Italian population-based cancer registries, covering 32% of the Italian population, able to provide at least 7 years of registration as of December 2009 and follow-up of vital status as of December 2013. The data were used to compute the limited-duration prevalence, in order to estimate the complete prevalence by means of the COMPREV software.

**Results:**

In 2010, 2,637,975 persons were estimated to live in Italy after a cancer diagnosis, 1.2 million men and 1.4 million women, or 4.6% of the Italian population. A quarter of male prevalent cases had prostate cancer (*n* = 305,044), while 42% of prevalent women had breast cancer (*n* = 604,841). More than 1.5 million people (2.7% of Italians) were alive since 5 or more years after diagnosis and 20% since ≥15 years. It is projected that, in 2020 in Italy, there will be 3.6 million prevalent cancer cases (+ 37% vs 2010). The largest 10-year increases are foreseen for prostate (+ 85%) and for thyroid cancers (+ 79%), and for long-term survivors diagnosed since 20 or more years (+ 45%). Among the population aged ≥75 years, 22% will have had a previous cancer diagnosis.

**Conclusions:**

The number of persons living after a cancer diagnosis is estimated to rise of approximately 3% per year in Italy. The availability of detailed estimates and projections of the complete prevalence are intended to help the implementation of guidelines aimed to enhance the long-term follow-up of cancer survivors and to contribute their rehabilitation needs.

## Background

Estimates of cancer prevalence are widely based on limited duration prevalence, including only patients living after a cancer diagnosis made in the previous 5 years [1, 2]. Prevalence, regardless of the time since diagnosis (i.e., complete prevalence), is less frequently estimated than limited duration prevalence [3–9]. Overall age-standardized cancer incidence and mortality rates have declined over the past 10 years in the majority of high income countries, whereas the complete prevalence has been consistently increasing in the early 2000s [3, 4, 6, 8, 10, 11]. Complete prevalence is generally measured in absolute numbers and proportions, i.e., not age-standardized. Thus, improved survival [12, 13] and population ageing (increasing absolute number of new cancer diagnoses) imply a progressive increase in tumour prevalence.

Cancer prevalence includes patients currently treated for cancer; those who have become cancer free, but still have a measurable excess risk of recurrence or death; and, finally, patients having death rates similar to those of the general population who can be considered “cured patients” [14]. Many of these individuals are possibly affected by physical, cognitive, and/or psychosocial limitations [15].

The aim of this study was to provide a description of the number of people living in Italy at January 1, 2010 after a cancer diagnosis, for all cancers combined and for a selection of cancer types by sex, age, and time since diagnosis. In addition, projections of cancer prevalence in Italy are presented up to the year 2020. Estimates and projections of complete tumour prevalence and characteristics of prevalent patients are necessary to help clinicians and health care planners in improving long-term care of patients and in allocating appropriately health care resources. Moreover, they may provide helpful information to a growing number of cancer patients or former patients.

## Methods

### Study design and data sources

This is a descriptive analysis of individual data collected during the period 1976-2009 from 27 population-based Italian cancer registries (i.e., 32% of the entire Italian population in 2010), which agreed to participate in the study and were able to provide at least 7 years of cancer registration as of December 31, 2009 (Appendix 1) and follow-up of vital status as of December 31, 2013. The Italian legislation identifies Cancer Registries as collectors of personal data for surveillance purposes without explicit individual consent. The approval of a research ethic committee is not required, since this descriptive study was conducted without any direct or indirect intervention on patients.

Prevalence for all malignant tumours (ICD-10: C00-C43, C45-C96) and 34 cancer types or their combinations were estimated and presented in this study for all age groups. Urinary bladder cancers with benign or uncertain behaviour, and in situ tumours were also included. Only non melanoma skin cancers (ICD-10 C44) were excluded. ICD-O-3 morphology codes were used to define specific subtypes.

### Statistical methods

The clinical and demographic characteristics of the persons registered with a diagnosis of cancers in the Italian CRs were used to estimate: 1) how many of them were still alive at January 1, 2010 regardless of time since diagnosis -i.e., complete prevalence count- by cancer type, sex, and age group; 2) the prevalence proportion in Italy at 2010 for each cancer type, by sex, and age; 3) the complete prevalence (count and proportion) at 1st January 2015 and 2020, overall and by time since diagnosis; and 4) describe the changing over time of these estimates.

For each cancer registry we computed the limited duration prevalence, i.e. the number of patients diagnosed in the period of the registration activity (between 7 and 34 years) at January 1, 2010, using the counting method implemented in SEER*Stat software [16]. This maximum limited duration prevalence was corrected, using the COMPREV software [17], by means of completeness index [18, 19], to estimate the total number of cancer patients alive, regardless of when they were diagnosed. Completeness indices were estimated by cancer type, sex, age, and time since diagnosis. Prevalence was computed as an absolute number, as well as a proportion per 100,000 residents people by cancer type, sex, age group, area of residence, and years since diagnosis. Patients with more than one primary cancer were included in the computation of prevalence for each cancer type or combination. In the analyses for all types combined, only the first cancer was considered. Completeness indices were obtained by statistical regression models of incidence and survival using data from 8 long-term registries (Appendix 1) with an available observation period of at least 18 years before 2010 [20, 21]. Relative survival and incidence functions were estimated by means of parametric models within the period 1985-2011 for survival and 1985-2009 for incidence. The survival model was a parametric cure model assuming that a proportion of individuals with cancer were bound to die (fatal cases) with a survival following a Weibull distribution, while the remaining proportion (cured fraction) had the same mortality rate as that of the general population with the same age and gender stratification [14, 20]. The parameters of the survival model were estimated by cancer type, sex, and age class (0-14, 15-44, 45-54, 55-64, 65-74, 75+ years) through the SAS procedure NLIN. A period effect was included on the hazard of dying of cancer. Incidence data were categorised according to cancer type, sex, five-year age group, and birth cohort (< 1899, 1900-1904,…, 2005-2009). A sixth degree polynomial age-cohort model of crude incidence rates was fitted through the SAS LOGISTIC procedure for each cancer type and sex [21].

Complete prevalence proportions were projected to 2020 by cancer type, sex, age, and registry, assuming that complete prevalence will follow a linear function, based on the trend of the last three calendar years (i.e., 2007-2009). This simplified assumption (linear and constant trend) may not be valid for long-term projections, but it is reasonable for short or medium-term (e.g., 10-year) ones. Other assumptions (e.g., log-linear models) were explored [4, 6], showing consistent results for common cancer types, but unstable projections for the rarest.

The absolute number of prevalent cases in Italy was obtained using proportions of prevalence estimates (age-, sex-, and cancer type-specific) from CRs included in this study, multiplied by the Italian national population by sex and age observed at January 1, 2010. Proportions projected to 2020 were thus multiplied to Italian population forecasted at January 1, 2020 [22].

## Results

### Prevalence estimates at 2010

In Italy in 2010, 2,637,975 persons were alive after a cancer diagnosis, corresponding to 4.6% of all the Italian population (Appendix 2). Prevalence proportions increase with age: 3.1% at age 45-54 years, 6.6% at 55-64 years, 12.1% at 65-74 years, and nearly 17% after age 75 years (Appendix 2) with differences by sex (Tables 1 and 2).

**Table 1:** Complete cancer prevalence by cancer type and age in Italian men at January 1, 2010

Cancer type	Prevalent cases									Prevalence proportion per 100,000 men							
	All ages	%	00-14	15-44	45-54	55-64	65-74	75-84	85+	All ages	00-14	15-44	45-54	55-64	65-74	75-84	85+
All types but skin non-melanoma	1,194,033		4844	84,172	87,091	198,505	363,932	357,051	98,439	4250	111	732	2079	5715	13,029	20,534	21,955
Upper aero-digestive tract	26,745	2.2%	19	1654	3320	6536	8063	5786	1367	100	0	15	84	199	311	337	313
Esophagus	3067	0.3%	0	54	252	722	1105	781	153	12	0	1	7	23	45	51	40
Stomach	45,970	3.8%	2	764	2583	6661	13,618	16,538	5802	158	0	6	58	180	470	926	1268
Small intestine	3384	0.3%	0	221	350	760	987	850	216	13	0	2	8	23	38	52	46
Colon, rectum, anus	185,532	15.5%	3	2718	8722	29,332	59,931	63,698	21,130	654	0	23	210	840	2108	3618	4682
Liver	17,454	1.5%	57	317	1539	3831	6347	4752	610	63	2	3	37	110	228	280	142
Biliary tract	4251	0.4%	0	70	238	713	1365	1443	421	15	0	0	6	20	47	80	103
Pancreas	5856	0.5%	3	198	598	1383	1876	1462	336	21	0	2	14	39	69	84	75
Larynx	44,810	3.8%	0	236	2105	8399	15,082	14,819	4169	160	0	2	51	240	540	854	965
Lung	63,048	5.3%	16	804	2771	11,014	22,765	21,682	3996	219	0	7	64	306	787	1229	890
Thymus, heart, mediastinum	2290	0.2%	42	384	435	548	516	331	33	7	1	3	9	14	18	18	9
Bone	4808	0.4%	152	1910	924	771	596	418	37	16	3	16	20	21	19	22	10
Skin melanoma	44,977	3.8%	21	6730	7411	9817	11,117	7867	2014	165	0	61	181	291	408	470	488
Mesothelioma	2090	0.2%	0	72	127	457	913	466	54	8	0	1	3	13	34	27	12
Kaposi sarcoma	5611	0.5%	3	567	658	864	1255	1498	766	21	0	5	17	26	46	90	174
Connective tissue	11,757	1.0%	226	2685	1696	2043	2459	2002	647	41	6	23	41	59	87	111	144
Penis	4285	0.4%	0	91	413	795	1309	1255	422	14	0	1	9	22	45	68	84
Prostate	305,044	25.5%	3	438	3387	34,764	112,958	122,376	31,118	1112	0	5	88	1048	4138	7143	6878
Testis	37,937	3.2%	86	17,116	8495	5349	3317	2389	1187	133	2	149	197	152	128	133	243
Kidney	62,815	5.3%	314	2842	5609	12,652	19,613	17,524	4262	226	7	25	134	364	703	1030	984
Bladder	192,611	16.1%	25	2802	8582	28,948	59,204	70,749	22,302	686	0	26	204	821	2104	4074	5053
Choroidal melanoma	1801	0.2%	0	115	209	365	484	519	109	7	0	1	6	11	18	30	25
Brain and central nervous system	16,110	1.3%	568	5391	2881	2930	2525	1423	391	54	13	46	65	81	82	78	66
Thyroid	25,512	2.1%	31	6428	5811	5876	4665	2351	349	89	1	56	137	165	166	136	80
Hodgkin lymphoma	27,821	2.3%	165	9685	5488	5229	4133	2684	437	95	4	83	129	139	141	148	99
Non-Hodgkin lymphoma	56,808	4.8%	629	8344	8754	11,691	13,802	11,185	2403	203	14	72	206	339	501	655	574
Leukemias	36,105	3.0%	1939	7620	4086	5656	8050	6703	2051	124	43	65	94	158	276	373	444
Multiple myeloma (plasma cell)	12,787	1.1%	0	326	1158	2636	4050	3680	938	45	0	3	27	75	143	215	207

**Table 2:** Complete cancer prevalence by cancer type and age in Italian women at January 1, 2010

Cancer type	Prevalent cases									Prevalence proportion per 100,000 women							
	All ages	%	00-14	15-44	45-54	55-64	65-74	75-84	85+	All ages	00-14	15-44	45-54	55-64	65-74	75-84	85+
All types but skin non-melanoma	1,443,942		3903	112,527	176,656	277,374	363,646	357,146	152,690	4836	93	988	4095	7496	11,243	13,994	14,500
Upper aero-digestive tract	15,433	1.1%	19	1562	1687	3156	3696	3624	1688	54	0	14	41	87	123	148	158
Esophagus	1125	0.1%	0	17	102	199	348	358	101	4	0	0	3	6	13	16	11
Stomach	35,537	2.5%	0	651	1896	3992	8619	12,953	7426	117	0	5	41	104	254	497	698
Small intestine	2597	0.2%	0	136	277	495	688	752	250	9	0	1	6	14	21	29	28
Colon, rectum, anus	171,847	11.9%	12	2754	8640	24,517	45,322	59,479	31,123	571	0	24	204	658	1377	2287	2901
Liver	7331	0.5%	61	258	371	943	2182	2926	589	25	1	2	9	25	68	114	58
Biliary tract	5565	0.4%	3	60	286	836	1517	1932	931	18	0	0	6	22	44	72	84
Pancreas	6271	0.4%	0	326	495	1239	1699	1733	780	21	0	3	11	33	55	68	69
Larynx	4407	0.3%	2	68	364	898	1211	1358	508	16	0	1	8	26	41	55	52
Lung	23,721	1.6%	5	611	2373	4933	7158	6662	1980	80	0	6	53	133	224	268	186
Thymus, heart, mediastinum	2212	0.2%	61	514	406	410	443	306	72	7	2	4	9	9	12	11	7
Bone	9124	0.6%	100	2259	2163	1950	1306	973	374	28	3	19	43	47	38	38	32
Skin melanoma	57,515	4.0%	30	10,718	9929	10,950	11,657	9953	4278	198	1	98	237	302	372	404	432
Mesothelioma	674	0.0%	0	18	68	148	224	174	42	2	0	0	2	4	8	8	5
Kaposi sarcoma	1990	0.1%	0	105	60	197	349	750	528	7	0	1	2	6	12	30	49
Connective tissue	9917	0.7%	203	1893	1399	1812	1890	1791	929	34	5	17	32	49	62	71	91
Breast	604,841	41.9%	0	26,663	82,068	128,514	165,456	142,658	59,483	2046	0	236	1906	3516	5164	5643	5751
Vagina and vulva	9689	0.7%	17	256	557	982	2377	3570	1931	32	0	2	13	27	71	137	183
Cervix uteri	58,879	4.1%	4	4321	8073	10,569	13,177	15,641	7093	193	0	38	184	280	397	591	675
Corpus uteri (endometrium)	103,321	7.2%	0	1490	5745	21,047	31,548	31,158	12,333	342	0	13	135	553	964	1198	1147
Ovary	45,620	3.2%	65	4058	6617	10,544	11,399	9729	3209	149	1	34	154	276	352	372	291
Kidney	35,250	2.4%	411	2369	2841	5290	9461	10,650	4229	122	9	21	68	149	293	436	418
Bladder	47,822	3.3%	6	1362	2562	6101	11,410	16,786	9594	164	0	12	62	172	359	676	897
Choroidal melanoma	1713	0.1%	0	149	210	294	445	414	202	6	0	1	4	9	14	18	21
Brain and central nervous system	23,145	1.6%	501	6210	3661	3565	3875	3978	1355	72	12	52	82	96	114	133	105
Thyroid	93,341	6.5%	68	22,813	21,805	21,597	16,956	8578	1524	307	2	199	498	571	521	356	153
Hodgkin lymphoma	20,433	1.4%	102	9116	3990	3104	2222	1401	498	67	2	79	93	84	67	58	43
Non-Hodgkin lymphoma	53,907	3.7%	262	5635	6626	10,917	13,615	12,731	4120	181	6	49	153	290	422	505	407
Leukemias	31,196	2.2%	1450	7445	3465	4400	5626	6067	2742	101	34	64	78	115	166	235	256
Multiple myeloma (plasma cell)	12,278	0.9%	0	217	887	2367	3611	3814	1382	41	0	2	22	64	112	150	124

Men living in Italy after a cancer diagnosis in 2010 were 1,194,033, corresponding to 4.3% (4250/100,000) of all Italian male population (Table 1). This proportion increased from less than 1% below the age of 45 years, to > 20% for men aged ≥75 years. The most frequent tumours in terms of prevalence were prostate (305,044 of prevalent cases at January, 1st 2010) representing 25.5% of all cases or 1.1% of all Italian men, followed by bladder (192,611 men, 16.1%) and colorectal (185,532 men, 15.5%) tumours.

Italian women living after a cancer diagnosis were 1,443,942 (Table 2), corresponding to 4.8% of all Italian women. Breast cancer represented 41.9% of all cancers (604,841), followed by colorectal cancers (171,847, 11.9% of all female prevalent cases, 0.6% of all Italian women) and by endometrial cancers (103,321, 7.2% and 0.3%, respectively). Notably, the fourth most frequent cancer type diagnosed in Italian prevalent women is thyroid (93,341 women, 6.5% of all female prevalent cases). Prevalent women were younger than men. Women aged 15-44 years living after a diagnosis represented 1% of the whole Italian population, they were 4% at ages 45-54 years, 7% at ages 55-64 years, 11% at ages 65-74 years, and 14% for women aged ≥75 years (Table 2).

**Fig. 1:**
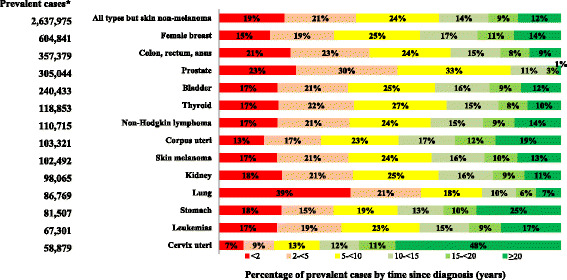
Complete prevalence by time since diagnosis for selected cancer types* in Italy at January 1, 2010. *Cancer types diagnosed in > 50,000 persons, sorted by number of cases

More than 1.5 million people (i.e., 2.7% of all Italian residents) were alive after ≥5 years since diagnosis. They were 60% of all prevalent cases, 64% of women and 55% of men. The distribution of prevalent cases by time since diagnosis depends on cancer type (Fig. 1). The percentage of prevalent cases diagnosed since less than 2 years varied from 39% for lung cancer patients to 15% for female breast and 7% for cervical cancer patients. Conversely, the percentage of prevalent cases diagnosed ≥15 years before was 59% for cervical cancer, 35% for stomach cancer and 31% for endometrial cancer, but only 4% for prostate and 13% for lung cancer patients. Notably, patients diagnosed ≥15 years before were 21% of all prevalent cases (16% among men and 25% among women).

### Prevalence projections for 2020

In 2020, there will be 3.6 million prevalent cancer cases in Italy (Table 3), 1.9 million women and 1.7 million men, with a 10-year increase of 37% (41 and 33% in men and women, respectively). In 2020, 2.6% of all Italian women (0.8 millions) will be alive after a breast cancer diagnosis and more than half a million patients (2.1% of all men) will be alive after a prostate cancer diagnosis (Table 3). The largest 10-year increases are foreseen for prostate (+ 85%) and for thyroid cancers (+ 79%, 212,863 cases), which will become the third most frequent prevalent cancer types among Italian women. A more than 50% increases are also expected in 2020 for prevalence after diagnosis of testicular cancer (63,395 patients) or skin melanoma (169,900). A limited change in prevalence (variations < 10%) is expected for ovary, larynx, and stomach, with cervical cancer being the only cancer type showing a decline in prevalence (− 13%) (Table 3).

**Table 3:** Projected complete prevalence (cases) at January 1,  2020 by sex and 10-year variations in Italy

	Prevalent cases			Variation (%)		
	2020			10-year period		
Cancer Type^a^	Men	Women	Total	Men	Women	Total
All types but skin non-melanoma	1687,049	1,922,086	3,609,135	41.3%	33.1%	36.8%
Upper aero-digestive tract	36,081	21,831	57,911	34.9%	41.5%	37.3%
Stomach	50,327	32,033	82,360	9.5%	−9.9%	1.0%
Colon, Rectum, Anus	280,277	233,245	513,522	51.1%	35.7%	43.7%
Liver	25,234	8531	33,765	44.6%	16.4%	36.2%
Larynx	47,015	6006	53,020	4.9%	36.3%	7.7%
Lung	77,159	40,657	117,816	22.4%	71.4%	35.8%
Skin Melanoma	80,069	89,831	169,900	78.0%	56.2%	65.8%
Connective Tissue	17,040	11,815	28,855	44.9%	19.1%	33.1%
Female Breast		834,154	834,154		37.9%	37.9%
Cervix Uteri		51,136	51,136		−13.2%	−13.2%
Corpus Uteri (endometrium)		122,553	122,553		18.6%	18.6%
Ovary		49,807	49,807		9.2%	9.2%
Prostate	563,960		563,960	84.9%		84.9%
Testis	63,395		63,395	67.1%		67.1%
Kidney	97,249	47,151	144,400	54.8%	33.8%	47.2%
Bladder	255,015	58,608	313,624	32.4%	22.6%	30.4%
Brain and central nervous system	23,505	29,314	52,819	45.9%	26.7%	34.6%
Thyroid	45,949	166,914	212,863	80.1%	78.8%	79.1%
Hodgkin Lymphoma	37,692	29,314	67,006	35.5%	43.5%	38.9%
Non- Hodgkin Lymphoma	82,780	73,584	156,364	45.7%	36.5%	41.2%
Leukaemias	45,880	39,100	84,980	27.1%	25.3%	26.3%
Multiple Myeloma	19,472	17,159	36,631	52.3%	39.8%	46.1%

^a^ Cancer types with more than 20,000 prevalent cases at 2010

**Table 4:** Projected complete prevalence at January 1, 2020 by sex and age groups in Italy ^a^

SEX, Cancer type	Prevalent cases					Prevalence proportion per 100,000			
	All ages	%	00-44	45-74	75+	All ages	00-44	45-74	75+
MEN and WOMEN									
All types but skin non-melanoma	3,609,135	100.0%	228,145	1,897,543	1,483,448	5731	726	16,383	21,657
Colon, rectum, anus	513,522	14.2%	4954	231,800	276,767	808	15	2080	3952
Skin melanoma	169,900	4.7%	24,038	101,180	44,682	271	76	857	673
Female breast	834,154	23.1%	29,758	498,614	305,781	2622	201	8215	7297
Corpus uteri (endometrium)	122,553	3.4%	1707	65,765	55,081	379	10	1104	1269
Prostate	563,960	15.6%	1174	255,514	307,272	2056	12	5634	12,343
Bladder	313,624	8.7%	4130	128,332	181,162	563	15	1323	2836
Thyroid	212,863	5.9%	41,112	145,562	26,189	309	127	1084	379
Non-Hodgkin lymphoma	156,364	4.3%	14,948	87,255	54,161	247	47	739	789
MEN									
All types but skin non-melanoma	1687,049	100%	95,056	834,967	757,026	5444	615	15,678	28,728
Colon, rectum, anus	280,277	16.6%	2250	135,206	142,821	902	13	2573	5267
Skin melanoma	80,069	4.7%	8760	50,437	20,872	256	57	898	815
Prostate	563,960	33.4%	1174	255,514	307,272	2056	12	5634	12,343
Bladder	255,015	15.1%	2636	106,086	146,294	958	20	2323	5932
Thyroid	45,949	2.7%	9141	31,444	5364	142	59	490	209
Non-Hodgkin lymphoma	82,780	4.9%	8959	49,513	24,309	271	58	871	946
WOMEN									
All types but skin non-melanoma	1,922,086	100%	133,089	1,062,575	726,422	5992	888	17,374	17,007
Colon, rectum, anus	233,245	12.1%	2704	96,594	133,947	720	17	1633	3105
Skin melanoma	89,831	4.7%	15,278	50,742	23,811	284	102	822	581
Breast	834,154	43.4%	29,758	498,614	305,781	2622	201	8215	7297
Corpus uteri (endometrium)	122,553	6.4%	1707	65,765	55,081	379	10	1104	1269
Bladder	58,608	3.0%	1494	22,246	34,868	195	10	405	859
Thyroid	166,914	8.7%	31,971	114,119	20,825	508	218	1761	516
Non-Hodgkin lymphoma	73,584	3.8%	5989	37,743	29,852	225	37	618	688

^a^ Most frequent cancer types are shown: Cancer types or combinations with > 100,000 prevalent cases

Nearly 22% (21,657/100,000) of population aged ≥75 years in 2020 will have had a previous cancer diagnosis (Table 4). Below 45 years of age, prevalent cases will be 228,145 (i.e., 0.8% of all cases, 726/100,000) and, in both sexes, the most frequent cancer type will be thyroid cancer, experienced by 31,971 women and 9141 men.

**Fig. 2:**
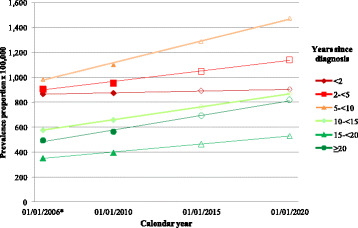
Complete cancer prevalence (proportions) in Italy from 2006 to 2020 by years since diagnosis. *Data for 2006 obtained from ref. 21. Filled symbols (e.g., •) represent estimated values, empty symbols (e.g., ο) represent projected values

Prevalent cases diagnosed within 2 years were the only group showing a negligible increase from 2010 to 2020 (+ 3% in the examined period), while a 19% increase was observed for cases diagnosed between 2 and 5 years before, 30-34% for cases diagnosed between 5 and 20 years earlier, and 45% increased for long-term survivors diagnosed ≥20 years before (Fig. 2).

## Discussion

In 2010, 2.6 million people were living in Italy after a cancer diagnosis and this number will reach 3.6 million in 2020, increasing from 4.6% to 5.7% (i.e., one out of 17 Italians) of the overall population. The estimated overall trend in the present decade in Italy (+ 3.2% per year) is comparable to that estimated in the same period in the USA (+ 2.8% per year) [5], UK (+ 3.3%) [4], and Switzerland (+ 2.5%) [6].

The expected 37% increase in the present decade in Italy will be more marked (i.e., nearly + 50%) among long-term survivors diagnosed ≥20 years before; they will be more than half a million in Italy (519,356), 14% of all prevalent cases (11% in men and 18% in women). Most of them can be considered as cured since they had already reached a similar life expectancy (i.e., death rates) of the corresponding general population [14].

A higher proportion of women (55%) than that of men emerged among prevalent cancer cases at 2010 in the present Italian study, in agreement with findings from most studies conducted in other countries [4–6, 9] but France (where 53% were men, 6.4% of the French population) [8]. In Italy, female breast cancer cases represented 23% of all prevalent cases, and affected the distribution of cancer prevalence by age. The thyroid cancer epidemic in Italy also contributed to an excess in females, below age 45 years thyroid cancer was the most frequent prevalent type in 2010 (29,340 men and women), and this number will substantially increase to more than 41,000 in 2020. It should be noted, however, that a large proportion of thyroid cancer incidence and prevalence may be affected by overdiagnosis; i.e., the detection of cancer cases that would not otherwise result in causing symptoms or deaths [23, 24].

An important role on variation of cancer prevalence is played by screening programmes, inducing a reduction of cervical and colorectal prevalent cancers cases, while early detection of breast and prostate cancers may inflate number of prevalent cases [25]. In particular, screening can prevent cervical cancer, with a consequent major effect on prevalence reduction, i.e., − 13% in 10 years in the present study.

Distribution of cancer prevalence by age is also noteworthy. In 2010, 37% of prevalent patients were 75 years or older (38% in men, 35% in women). In this age group, they will reach 41% in 2020, with more than 20% of men and 14% of women will have experienced a previous cancer diagnosis. These proportions were similar to those reported by other studies, showing also that elderly cancer patients had more severe comorbidity conditions than non cancer patients [26].

At the opposite end of the age spectrum, 8% of Italian prevalent cases were younger than 44 years of age and 10% were aged 45–54 years. It has been recently estimated that 44,135 persons living in Italy in 2010 had had a cancer diagnosis during childhood [27]; they represented 0.07% of the Italian population and 1.7% of prevalent cases diagnosed at any age. In similar studies conducted in the USA [28], a substantial proportion of morbidities emerged in childhood cancer patients several years after diagnosis, and there is growing awareness on potentially long-term risks affecting the survivors’ future physical, cognitive, and/or psychosocial health [29]. The impact of a cancer diagnosis is rather different between younger and older survivors, the first facing more pronounced socio-economic consequences [30, 31], as well as psychosocial impairments in fertility and sexuality [32, 33].

We acknowledge the several limitations of our analyses. First, data from Italian cancer registries (AIRTUM) included one third of the Italian population in 2010 and the representativeness for the national prevalence estimates may be questionable [34]. To overcome this issue, we adjusted estimated proportions in cancer registry areas for the age distribution of the whole Italian population. Moreover, since cancer registries have been active in Italy from a relatively recent time period, the complete prevalence has been estimated through statistical models. Notably, the validation of complete prevalence estimation by means of COMPREV method in Italy and elsewhere [19] is reasonably reassuring. In particular, the validation of COMPREV method shows negligible (i.e., < 5%) differences, when comparing observed prevalence for cancer registries with ≥30 years of observation and estimated prevalence using complete indexes applied to the same registries and truncated data [21, page 34].

On the other hand, the strengths of this population-based study are represented by the size of the study population, which included nearly 1.7 million incident cancer cases, and its long-term follow-up, more than a half of these cases were followed-up for > 20 years post diagnosis. In addition, data and period used were updated in the present study (see Appendix 1), including an additional number of years of observation and follow-up, in comparison with previous studies on the same topic [21].

The accuracy of future projections of prevalence is necessarily uncertain and lies on statistical models based on assumptions reflecting unknown evolution of incidence, survival, and demographic changes. This may also affect comparisons with trends reported in other countries, obtained using different assumptions and statistical models [4, 6, 26]. In our medium-term projections, the hypothesis that complete prevalence at 2020 can be predicted by a linear function of calendar year as regressor variable is supported by empirical evidence, at least for all cancer types combined and for most frequent cancer types, consistently showing an approximate linear trend in recent years [5, 21]. Notably, the use of a longer period (5 calendar years) to estimate linear slope did not materially modify the estimates.

Detailed estimates and projections of numbers of persons living after different cancer diagnoses are particularly relevant for policy makers to better plan health care resource allocation and meet cancer patients needs, including not only initial treatment, but also rehabilitation and long-term surveillance. However, to date, guidelines pertaining to survivorship care have been largely based on consensus rather than on empirical evidence [35–37].

In the USA, the main driver of cancer costs growth is population ageing, with an overall increase of 27% by the year 2020 from 2010 levels [38]. The largest increase in expenditures is attributable to the continuing phase of care (i.e., > 1-year post-diagnosis and > 1 year from death) for prostate and female breast cancer, with 42 and 32% increase respectively [38]. Although health care costs in the continuing phase of care is lower than in the first course of treatment (first year since diagnosis) and in the last year of life, the large number of survivors in the continuing phase of care is driving most of healthcare resources. Similar findings, on the distribution of cancer burden by phase of care, are expected in Italy [39].

## Conclusions

The availability of reliable and accurate estimates of complete prevalence and predictions of the rising tide of people living after cancer diagnosis may be helpful not only to epidemiologists and health-care planners, but also to clinicians in developing guidelines to enhance and standardize the long-term follow-up of cancer survivors. Furthermore, these estimates are intended for patients to help recovering social activities and supporting rehabilitation demands.

## Appendix 1

**Table:** Population and incident cases in Italian cancer registries with ≥7 years of registration in period 1976-2009

CANCER REGISTRY	Period of activity		Population at January 1st 2010	Incident cases up to 2009^a^
	Period of registration	Years included to 2009	(per 1000)	
Alto Adige - Sudtirol	1995–2010	15	494	37,119
Biella	1995–2010	15	185	20,362
Catania-Messina	2003–2011	7	1727	58,753
Catanzaro	2003–2009	7	230	7755
Como	2003–2011	7	577	24,963
Ferrara^b^	1991–2011	19	354	50,925
Friuli Venezia Giulia	1995–2010	15	1219	128,738
Genova^b^	1986–2009	24	592	112,812
Latina	1996–2011	14	531	32,330
Mantova	1999–2010	11	404	27,541
Milano	1999–2010	11	1215	103,283
Modena^b^	1988–2011	22	676	84,155
Napoli	1996–2011	14	561	28,250
Nuoro	2003–2011	7	219	7889
Palermo	2003–2011	7	1239	40,926
Parma^b^	1978–2011	32	420	80,744
Ragusa^b^	1981–2011	29	303	31,283
Reggio Emilia	1996–2011	14	508	41,379
Romagna	1993–2011	17	1058	119,458
Salerno	1996–2009	14	1089	63,293
Sassari^b^	1992–2011	18	467	37,988
Siracusa	1999–2011	11	400	18,927
Sondrio	1998–2011	12	181	13,003
Trapani	2002–2009	8	429	15,591
Umbria	1994–2011	16	875	85,138
Varese^b^	1976-2011	34	860	137,184
Veneto^b^	1990–2009	20	2097	245,898
All CRs			18,909	1,655,687
Italy			59,190	

^a^ All types but skin non-melanoma

^b^ CRs included to estimate model-based incidence and survival (47% of all cancer cases)

## Appendix 2

**Table:** Complete cancer prevalence (cases and proportion) by cancer type and age at prevalence in Italian men and women at  January 1, 2010

Cancer type	Prevalent cases									Prevalence proportion × 100,000^a^							
	All ages	%	00-14	15-44	45-54	55-64	65-74	75-84	85+	All ages	00-14	15-44	45-54	55-64	65-74	75-84	85+
All types but skin non-melanoma	2,637,975		8747	196,699	263,746	475,879	727,578	714,197	251,129	4552	102	859	3103	6635	12,068	16,620	16,700
Upper aero-digestive tract	42,178	1.6%	38	3216	5007	9692	11,759	9410	3055	76	0	14	62	142	209	224	204
Esophagus	4192	0.2%	–	71	354	921	1453	1139	254	8	–	0	5	15	28	30	20
Stomach	81,507	3.1%	2	1415	4479	10,654	22,237	29,491	13,229	137	0	6	49	141	354	669	866
Small intestine	5981	0.2%	–	356	627	1255	1675	1602	466	11	–	2	7	18	29	38	33
Colon, rectum, anus	357,379	13.5%	15	5472	17,362	53,849	105,252	123,177	52,253	611	0	23	207	746	1715	2821	3426
Liver	24,785	0.9%	119	575	1910	4774	8529	7678	1199	43	1	3	23	66	142	180	83
Biliary tract	9816	0.4%	3	131	524	1549	2882	3375	1352	16	0	0	6	21	45	75	90
Pancreas	12,128	0.5%	3	524	1093	2622	3575	3194	1116	21	0	2	12	36	61	75	71
Larynx	49,217	1.9%	2	304	2469	9297	16,293	16,177	4676	86	0	1	29	129	271	376	321
Lung	86,769	3.3%	21	1415	5144	15,947	29,923	28,343	5976	147	0	6	58	216	484	654	394
Thymus, heart, mediastinum	4501	0.2%	103	898	842	958	959	636	105	7	1	3	9	11	15	14	8
Bone	13,932	0.5%	251	4168	3087	2721	1902	1391	411	22	3	17	32	34	29	32	26
Skin melanoma	102,492	3.9%	52	17,448	17,339	20,767	22,774	17,820	6292	182	1	80	210	296	388	430	448
Mesothelioma	2763	0.1%	–	90	195	605	1137	640	96	5	–	0	2	8	20	15	7
Kaposi sarcoma	7601	0.3%	3	672	718	1061	1605	2248	1294	14	0	3	9	16	27	54	86
Connective tissue	21,674	0.8%	429	4578	3095	3855	4349	3793	1576	37	6	20	37	54	74	87	107
Female breast	604,841	22.9%	–	26,663	82,068	128,514	165,456	142,658	59,483	2046	–	236	1906	3516	5164	5643	5751
Vagina and vulva	9689	0.4%	17	256	557	982	2377	3570	1931	32	0	2	13	27	71	137	183
Cervix uteri	58,879	2.2%	4	4321	8073	10,569	13,177	15,641	7093	193	0	38	184	280	397	591	675
Corpus uteri (endometrium)	103,321	3.9%	–	1490	5745	21,047	31,548	31,158	12,333	342	–	13	135	553	964	1198	1147
Ovary	45,620	1.7%	65	4058	6617	10,544	11,399	9729	3209	149	1	34	154	276	352	372	291
Penis	4285	0.2%	–	91	413	795	1309	1255	422	14	–	1	9	22	45	68	84
Prostate	305,044	11.6%	3	438	3387	34,764	112,958	122,376	31,118	1112	0	5	88	1048	4138	7143	6878
Testis	37,937	1.4%	86	17,116	8495	5349	3317	2389	1187	133	2	149	197	152	128	133	243
Kidney	98,065	3.7%	725	5211	8450	17,941	29,073	28,174	8491	172	8	23	101	253	483	674	585
Bladder	240,433	9.1%	31	4164	11,144	35,049	70,614	87,535	31,896	416	0	19	132	485	1165	2041	2123
Choroidal melanoma	3514	0.1%	–	264	419	658	929	933	311	6	–	1	5	10	16	23	22
Brain and central nervous system	39,255	1.5%	1069	11,602	6542	6495	6400	5401	1746	63	13	49	74	89	99	111	94
Thyroid	118,853	4.5%	99	29,241	27,617	27,474	21,622	10,929	1872	202	1	127	321	375	357	268	131
Hodgkin lymphoma	48,254	1.8%	266	18,801	9478	8333	6355	4085	935	81	3	81	111	110	101	94	59
Non-Hodgkin lymphoma	110,715	4.2%	891	13,979	15,380	22,608	27,417	23,916	6523	191	10	61	179	314	458	565	456
Leukemias	67,301	2.6%	3389	15,065	7551	10,057	13,676	12,770	4793	112	39	64	86	136	217	291	312
Multiple myeloma (plasma cell)	25,066	1.0%	–	542	2044	5003	7662	7494	2320	43	–	2	24	69	126	176	149

^a^ For sex-specific types proportions were also sex-specific
